# *In silico* Platform for Prediction of N-, O- and C-Glycosites in Eukaryotic Protein Sequences

**DOI:** 10.1371/journal.pone.0067008

**Published:** 2013-06-28

**Authors:** Jagat Singh Chauhan, Alka Rao, Gajendra P. S. Raghava

**Affiliations:** 1 Bioinformatic Centre, Institute of Microbial Technology, Chandigarh, India; 2 Protein Science and Engineering, Institute of Microbial Technology, Chandigarh, India; Universita' di Padova, Italy

## Abstract

Glycosylation is one of the most abundant and an important post-translational modification of proteins. Glycosylated proteins (glycoproteins) are involved in various cellular biological functions like protein folding, cell-cell interactions, cell recognition and host-pathogen interactions. A large number of eukaryotic glycoproteins also have therapeutic and potential technology applications. Therefore, characterization and analysis of glycosites (glycosylated residues) in these proteins is of great interest to biologists. In order to cater these needs a number of *in silico* tools have been developed over the years, however, a need to get even better prediction tools remains. Therefore, in this study we have developed a new webserver GlycoEP for more accurate prediction of N-linked, O-linked and C-linked glycosites in eukaryotic glycoproteins using two larger datasets, namely, standard and advanced datasets. In case of standard datasets no two glycosylated proteins are more similar than 40%; advanced datasets are highly non-redundant where no two glycosites’ patterns (as defined in methods) have more than 60% similarity. Further, based on our results with several algorihtms developed using different machine-learning techniques, we found Support Vector Machine (SVM) as optimum tool to develop glycosite prediction models. Accordingly, using our more stringent and non-redundant advanced datasets, the SVM based models developed in this study achieved a prediction accuracy of 84.26%, 86.87% and 91.43% with corresponding MCC of 0.54, 0.20 and 0.78, for N-, O- and C-linked glycosites, respectively. The best performing models trained on advanced datasets were then implemented as a user-friendly web server GlycoEP (http://www.imtech.res.in/raghava/glycoep/). Additionally, this server provides prediction models developed on standard datasets and allows users to scan sequons in input protein sequences.

## Introduction

Along with DNA and RNA, proteins form the quintessential molecules of life in a cell. Proteins are the workhorse performing a variety of jobs inside a cell. There are a number of strategies by which a protein and its function can be further influenced or fine-tuned inside a cell. Post-translational modification of proteins is one of such strategies. Among all such known modifications, glycosylation is the most abundant post-translational modification of proteins whereby a carbohydrate moiety termed as glycan moiety is attached covalently to the peptide backbone of a protein in different ways to give rise to a glycoprotein glycoconjugate.The impact of glycosylation on proteins may range from very subtle to the ones that are crucial for the development, growth, function, or survival of an organism. Glycoproteins have been implicated in various biological roles namely, involvement in protein shape/structure maintenance, protein refolding regulations, plasma membrane rigidity, enzymatic activities, locomotion, immunogenicity, antigenicity, pathogenecity and most importantly cell surface properties that are crucial for host- pathogen interactions and pathogen resistance to host complement killing [1], [2], [3], [4], [5]. In nature, dedicated enzymes named as glycosyltransferases (GT) carry out protein glycosylation events in a cell. Based on the linkage between the amino acid and the sugar, the process of glycosylation is categorized into five types in eukaryotes: N-linked, O-linked, C-linked, P-linked and G-linked within which most common are N- and O-linked glycosylation. GPI (Glycosylphosphatidylinisotol) anchors attachment (G-linked or) and phospho-glycans linking through the Phosphate of a Phosphoserine (P-linked) are generally rare.

N-linked glycosylation is characterized by the addition of a glycan moiety (GlcNAc normally) to a Nitrogen atom, usually the N4 of Asparagine (Asn) residues by an oligosaccharyltransferase (OST) that specifically recognizes a consensus sequence (sequon) Asn-X-Ser/Thr, where X is any amino acid except Proline (Pro) [6], [7]. The process occurs in endoplasmic reticulum and helps in protein folding and its trafficking, in eukaryotes.

O-linked glycosylation occurs in the Golgi apparatus following N-linked glycosylation. Free hydroxyl group containing amino acid residues that includes Serine (Ser), Threonine (Thr) and to some extent, hydroxylproline and hydroxylysine are the possible sites for O-glycan attachment. In comparison to N-linked glycosylation, the O-linked glycosylation is not yet known to occur on any consensus sequence in eukaryotes, though presence of Proline and beta sheet conformation around O-glycosites is suggested to be favored here [8], [9].

The C-linked glycosylation is comparatively rare event and in this the glycan is found attached at carbon of the first Tryptophan (Trp) residue in the consensus sequence W-X-X-W or W-X-X-C or W-X-X-F (where X is any amino acid) [10]. As N- and C-linked glycosylations take place in the Endoplasmic Reticulum and/or the Golgi apparatus, only extracellular or secreted proteins get glycosylated in eukaryotes Whereas, both intracellular and extracellular proteins can get glycosylated by O- linked glycosylation.

Protein glycosylation in prokaryotes is rather recent knowledge. In this context, though there are many similarities in prokaryotes and eukaryotes, protein glycosylation could be substantially different and/or much more versatile in prokaryotes in terms of mechanisms and enzymes used, glycans attached, donor acceptor preferences as well in the presence of novel glycosites (Cysteine-linked) and sequons [11], [12], [13]. In our recently published studies, we too had analysed, the experimentally verified glycosites of prokaryotic glycosites vis-a vis corresponding eukaryotic glycosites for the sequence/structural contexts around glycosylated residues to conclude that the two differ significantly (discussed in the sections Introduction, Results and Figure, S1, S2 and S3 of reference [12]. The same was highlighted by Dell and co-workers in the past [13]. Further, it suggests that prediction softwares should be developed exclusively and independently for prokaryotic and eukaryotic glycosites**.** Nonetheless, with more than 70% of human therapeutic proteins as glycoproteins [14] and expensive, time consuming process of experimental annotation of glycoproteins and glycosites in proteins, better *in-silico* tools remain desirable [15].

At present several methods for prediction of glycosites are available for eukaryotic glycosites but most of these methods have been developed using more or less identical and highly redundant datasets. These datasets may also contain (ex. O-glycbase) prokaryotic glycosites albeit not many. Most of the existing glycosylation prediction servers cater either one or maximum two glycosylation-type predictions, at one platform/web-server. For example prediction of sites with GPI modification has been addressed in reference [16], prediction of only mucin type of glycosylation has been made available through NetOglyc [17], [18] and Oglyc [19]. Similarly, NetNglyc for N-linked prediction server are widely used in glycosite prediction in mammals and human proteome [20]. A detailed account of these existing methods can also be obtained from already published review [21]. Currently only one open access webserver titled EnsembleGly [22] provides for prediction of N-, O- and C-linked glycosylation at one place. Another webserver GPP [23] addresses only N and O-linked glycosites’ prediction. Further the datasets used to develop these methods were also very small due to limited sequences availablility at that time. Therefore, in this study we aimed to develop new and better prediction models for prediction of N-, O- and C-linked glycosites in eukaryotic protein sequences. In this study new and a larger dataset of (experimentally verified) 2004 eukaryotic glycoproteins has been created from SWISS-PROT_June 2011 release. This dataset was then further improved in to standard and advanced datasets by redundancy reduction at the level of protein sequence and subsequently at the level of glycosite patterns. Using these non-redundant datasets, several algorithms/models were developed, tested and finally the best performing models are implemented for open access at web server GlycoEP.

## Materials and Methods

### Datasets

All datasets used in this study, contain eukaryotic glycoprotein (and glycosites) only. The primary glycoprotein dataset used in this study was extracted from SWISS-PROT June 2011 release by using key name “GLYCOSYLATION” and excluding all “PROBABLE”, “BY SIMILIARITY” and “PREDICTED” entries. This primary redundant datasets (1797 N-linked, 193 O-linked and 14 C-linked proteins) were than subjected to redundancy reduction of different stringency to derive following datasets for further study:

#### Standard and independent datasets

**Figure 1:**
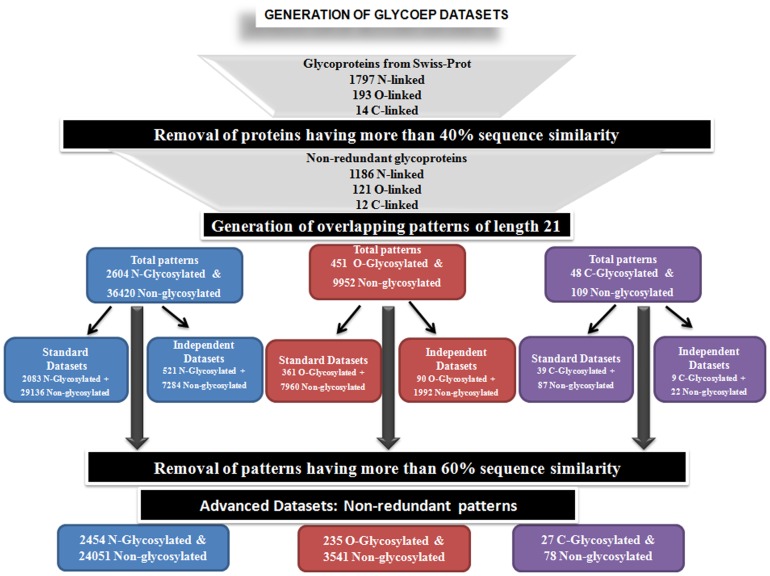
Flowchart showing process for creating various datasets used for developing GlycoEP models.

**Figure 2:**
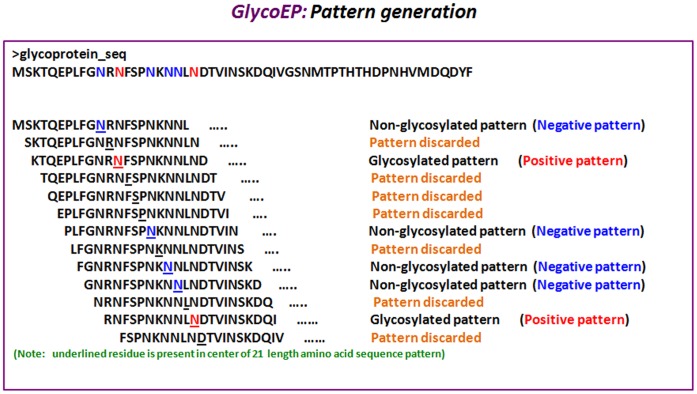
The process of creating of overlapping patterns in a glycoproteins and assigning glycosylated and non-glycosylated patterns.

Using CD-Hit program with cut-off 40% we got non-redundant standard datasets of 1186 N-, 121 O-, 12 C-linked proteins, where no two proteins are more similar than 40% at the level of (full-length) protein sequence (Figure 1). From these protein sequences overlapping patterns of residue length 21 were generated where glycosylated residue is the central residue of the pattern (Figure 2). Accordingly, we got total 2604 N-linked glycosites (with corresponding 36420 non-glycosylated/negative N-linked sites), 451 O-linked glycosites (with corresponding 9952 non-glycosylated/negative sites) and 48 C-linked glycosites (with corresponding 109 non-glycosylated/negative sites). These patterns were further divided in two parts at a ratio of 4∶1, in order to create standard and independent (blind/unseen) datasets. Thus our standard datsets contain 2083 N-linked glycosites (with corresponding 29136 non-glycosylated/negative N-linked sites), 361 O-linked glycosites (with corresponding 7960 non-glycosylated/negative sites) and 39 C-linked glycosites (with corresponding 87 non-glycosylated/negative sites). Similarly, our independent datasets contain 521 N-linked glycosites (with corresponding 7284 non-glycosylated/negative N-linked sites), 90 O-linked glycosites (with corresponding 1992 non-glycosylated/negative sites) and 9 C-linked glycosites (with corresponding 22 non-glycosylated/negative sites).

#### Advanced datasets

Our standard datasets were generated from non-redundant proteins in order to minimize similarity between testing (independent) and training (standard) datasets. As the similarity was removed at the protein level, redundancy still was present in 21 residues long overlapping patterns that were generated from these proteins as explained above as well as in “Patterns and profile construction” section. As to get a robust prediction, a highly non-redundant dataset is always a pre-requisite, we further carried out a redundancy reduction at the pattern level to obtain a highly non-redundant advanced dataset where no two patterns are more similar than 60% [24]. The advance datasets thus contains 2454 N-linked, 235 O-linked, 27 C-linked glycosylated/positive patterns with corresponding non-glycosylated/negative patterns: 24051 for N-linked, 3541 for O-linked and 78 for C-linked respectively (Table 1). These highly non-redundant advanced datasets are used for developing advanced moled for predicted glycosylation (Figure 1).

**Table 1:** Non-redundant glycosylated and non-glycosylated (positive+negative) patterns at different level of similarity cut-off.

Redundancy cut-off	Number of total patterns (glycosylated plus non-glycosylated)		
	N-linked (Positive+Negative)	O-linked (Positive+Negative)	C-linked (Positive +Negative)
**Standard dataset**	39024 = (2604+36420)	10403 = (451+9952)	157 = (48+109)
100%	39019 = (2604+36415)	10371 = (451+9920)	157 = (48+109)
90%	35293 = (2588+32705	7314 = (339+6975)	150 = (48+102)
80%	32245 = (2549+29696)	5669 = (289+5380)	116 = (32+84)
70%	29376 = (2506+26870)	4566 = (258+4308)	106 = (27+79)
60%	26505 = (2454+24051)	3776 = (235+3541)	105 = (27+78)
50%	23076 = (2361+20715)	3234 = (214+3020)	99 = (23+7)
40%	10102 = (1599+8503)	2390 = (174+2216)	90 = (16+74)

### Machine Learning Tools

In the present study, we have attempted to trainWeka classifiers namely**,** SMO, libsvm, Random Forest, LMT, Bayesian network, naive Bayes and RBF Network as well as SVM. Weka is a widely accepted machine-learning toolkit in bioinformatics implemented in Java [25]. The data for Weka is represented in ARFF (attribute-relation function format) format that consists of a list of all instances, with the attribute values for each instance being separated by commas. The results from the Weka consists of a confusion matrix for both the training and testing set showing the number of instances of each class that have been assigned to each class. Similarly, SVM is based on the structural risk minimization principle from statistical learning theory. In SVM classifier the parameters and kernel functions like linear, polynomial, radial basis function, sigmoid can be adjusted easily [26]. In this study, we have used linear, polynomial and RBF kernel and learning option −g (1.0.0.1,0.01,0.001,0.0001,0.00001), −C parameter (ranges from 1to10) and −j parameter range from 1to 4 and observed that RBF kernel performed better than the others. So finally we have optimized all SVM learning models using RBF kernel [27].

### Patterns and Profile Construction

Overlapping patterns of residue length 21 were generated for each glycosite (as shown in Figure 2). The patterns thus generated were termed as positives in cases where central residue is glycosylated and negatives if the same is unglycosylated [12], [28], [29]. To generate a pattern corresponding to the terminal residues in a protein sequence, (L-1)/2 dummy residue "X" is appended at both the termini of the protein (where L is the length of pattern).

### Binary Profile of Patterns (BPP)

Fixed length amino acid patterns were converted into binary values [12], [28]. Each residue of patterns was represented by a vector of dimension 21 (e.g. Ala by 1,0,0,0,0,0,0,0,0,0,0,0,0,0,0,0,0,0,0,0,0; Cys by 0,1,0,0,0,0,0,0,0,0,0,0,0,0,0,0,0,0,0,0,0), which contains 20 amino acids and one dummy amino acid "X".

### Composition Profile of Patterns (CPP)

Composition profile of patterns is the fraction of each amino acid in a fixed length sequence pattern. In case of amino acid composition, variable length protein sequences were represented by fixed length patterns of 20 residues.

### PSSM Profile of Patterns (PPP)

We have also attempted to use PSI-BLAST generated PSSM profile as an input feature for the training of SVM model. The PSSM for each sequence was generated by PSI-BLAST search against of SWISS-PROT. After three iterations with cut-off E-value of 0.001, it generated a PSSM having the highest score as a part of the prediction process. The PSSM matrix contains 20×M elements, where M is the length of the target sequence. The PSSM contains probability of occurrence of each type of amino acid at each residue position of fixed length sequence patterns.

### Secondary Structure Information

Three state secondary structure (Coil, Helix and strand) information that were obtained using PSIPRED [30] were used as input in this study. The PSIPRED provided three secondary structure states for each residue of protein with probability.

### Surface Accessibility Information

The accessible surface area (ASA) is the surface area of a protein that is accessible to another protein or ligand. The predicted average accessible surface area values of each amino acid were calculated from sarpred [31].

Based on the understanding that the glycosylation (especially O-glycosylation) may occur preferentially at surface accessible or exposed regions/residues in a protein and that it may also be governed/influenced by certain preferred secondary structural features like beta sheets or turns/loops, we have attempted to include two additional prediction features namely, SS (predicted secondary structure information) and ASA (predicted accessible surface area) in the overall prediction schema. These features have been used as input features in different combinations with BPP and CPP profiles of the sequences and best ones are implemented as an optional method for the users.

### Training and Evaluation

In this study, we have used 5-fold cross-validation procedure to train and develop the prediction methods, where five subsets have been constructed randomly from the datasets as described in previous studies [12], [28], [29]. The models have been trained on four sets and the performance is measured on the remaining fifth set. This process is repeated five times in such a way that each set is used once for testing. The final performance is obtained by averaging the performances of all five sets. Standard deviations for iterative five fold training cycles are calculated and reported with corresponding avergae performance of each method for better judgement on performance stability of the method.

The performances of the different methods have been evaluated using parameters namely, sensitivity, specificity, accuracy and MCC (threshold- dependent parameters). Threshold selection is important criteria for checking the consistency of prediction result. In our study, the threshold varied in the range of –1 to +1, normally “0″ was selected as as default threshold that gave balance between sensitivity and specificity. Receiver Operating Characteristic (ROC, using R-package at http://www.r-project.org/) plots (threshold- independent parameter) were drawn between TP rate and FP rate and Area under Curve (AUC values) is calculated, accordingly.

## Results and Discussion

### Probability of Amino Acids Around Glycosites

In order to decipher the significant patterns the probability/frequency of specific amino acid residues in and around a glycosite can be derived from weblogos using glycosylated patterns of fixed sequence length. Requirement of a consensus sequence comprising of a tripeptide motif N-X-S/T (where X is any amino acid except Pro) is well established for N-linked glycosylation. Similarly, for C- linked glycosylation a known consensus sequence is W-X-X-W or W-X-X-C or W-X-X-F (where X is any amino acid). However, in case of O-linked glycosites no such defined sequence has thus far been identified in eukaryotic glycoproteins.

The amino acid frequency weblogos for sequences encompassing ten residues on either sides of eukaryotic glycosites in our dataset of 2604 N-linked glycosites (derived from 1186 glycoproteins) suggests that above mentioned consensus sequences are almost always true in case of N-linked and C-linked glycosites. However, exceptions some of which are reported previously are listed in Table S1 where an N-P-S/T, N-X-C and such other rare sequence motifs are found glycosylated at Asn residue. Similarly, weblogos developed from 456 O-linked glycosites (derived from 121 eukaryotic glycoproteins) are also in accordance with known preponderance of Ser and Ala (Alanine) residues around O-glycosites of eukaryotic (mucin type) glycoproteins. Preference for Pro at positions −3, +3 and +1 around glycosylated Ser/Thr (if at position 0) was also observed in our datasets. Additionally, we observed higher frequency of Pro at −6 positions and Gly (Glycine) at +1 position in proteins that are glycosylated at Thr residues (Figure S1, S2, S3). Besides this first we have analysed few glycoproteins to check motif based detection of glycosylated sites in N-linked sequon and we observed that sequon (motif) is easily detected by our method (Table 2).

**Table 2:** The performance of sequon (motifs) detection in N-linked glycosylation using five independent glycoproteins on GlycoEP server.

Glycoprotein IDs	Total Asparagine residuesin whole sequnec	Total detection of N-linked sequonin sequence (NXS/T)	Actual N-linked sequon
P28825	41	10	9
P81447	8	1	1
P06756	50	13	4
P31809	47	16	8
P01833	39	7	7

### Spread of Glycosites Across Protein Lengths

Analysis of 2604 N-linked glycosites in eukaryotic glycoproteins suggest that N-linked glycosites are spread out across the protein sequence length and the probability of N-linked glycosylation was observed poorest in first 5% (N terminal) of sequence length and last 30% of sequence length toward C terminus of protein. Similar analysis of spread of glycosites across O-linked glycoproteins suggested that the most likely position of O-linked glycosylation in eukaryotic glycoproteins is at around N terminal 6–10% of the sequence length followed by another high probability region at around middle 46–50% of the sequence length (Figure S4).

### Comparision of Performance of Weka and SVM Classifiers

Using balanced Unique Glycoprotein datasets we have trained several Weka classifiers (as explained above) as well as SVM classifier for the prediction of N-, O- and C- glycosites in eukaryotic protein sequences. The performances of these classifiers using five fold cross validation, were then compared with each other. The detailed results are presented in Table S2, S3, S4, S5, S6, S7, S8, S9, S10. From this comparision, we found overall performance of SVM classifier the best among all tried classifiers, in our hands. Therefore, the further optimization were pursued only with SVM classifier.

### Prediction of Glycosites Using SVM on Standard Datasets

**Figure 3:**
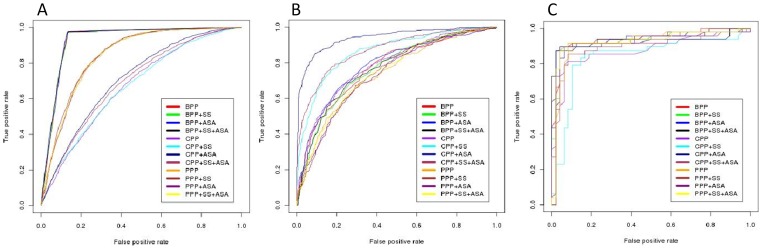
Performances of various models on standard datasets in term of ROC, for N-, O- and C-linked glycosites (Panel A, B and C, respectively) in eukaryotic proteins.

Almost all well-known and existing glycosite prediction methods have been developed using only sequence based non-redundant datasets (standard datasets). Therefore, we first developed our models using standard datasets for predicting N- O- and C-linked glycosites and then have compared them with some of these already known prediction programmes (discussed below). Using our models for N- and C-linked glycosites a prediction accuracy of 87.95% and 91.08% was achieved with corresponding MCC of 0.54, and 0.79, respectively and with BPP as input feature. However, O-glycosites’ prediction was best done using CPP based models, where the models achieved an accuracy of 84.08% and an MCC of 0.32. Further inclusion of ASA as an additional input feature with CPP improved O-glycosites’ prediction to an accuracy of 93.12% though the same could not be achieved for N- and C-glycosites (Table S11, S12 S13). Presence of a defined and conserved sequon feature for N-glycosites (but none for O-glycosites) could be one explanation for this result. The ROC plots of the best optimized prediction model using standard datasets are presented in Figure 3. Further, as in most functional residues prediction method there is always a dissimilarity in the ratio of positive and negative instances of the training data, where negative instances are usually much higher than the positive instances. Therefore, in this study we have primarily trained all our methods using such standard datasets (explained under section Datasets). It is well known fact that machine learning techniques learn best on balanced datasets, thus we also trained and tested our models using the balanced datasets containing equal number of glycosylated (positive) and unglycosylated (negative) sites. The performances of these models trained on balanced datasets is shown in following Table S14, S15, S16. In our study, performances of models trained on standard datasets is at par with the performance obtained with related balanced datasets.

### Development and Implementation of Advanced Models

Specificity in glycosylation event is largely governed by the specific sequon/sequence motifs/sequence contexts/sequence patterns. Therefore, we have developed advanced models using a dataset where high redundancy reduction has been executed in the glycosite patterns (sequence windows) to obtain a non-redundant advanced datasets (though smaller than standard dataset) for better class divide between negative and positive patterns. The advantages of this approach has been discussed previously in [24] and the approach has been employed successfully in improving prediction of PTM’s associated with lysine residue in proteins [32]. We developed SVM models on advanced datasets and achieved maximum accuracy value 84.24%, 86.87% and 91.43% for N-, O- and C-linked glycosylation, respectively (with corresponding maximum MCC values of 0.54, 0.20 and 0.78). We have also developed SVM model using balanced patterns of advanced datasets for N-, O- and C-linked glycosylation and achieved maximum accuracy of 92.26%, 62.77% and 79.82% with MCC of 0.85, 0.27 and 0.66, respectively. We term these models as Advanced GlycoEP models for these provide biologically more meaningful and also robust predictions in comparision with standard models and other similar methods discussed above. It was observed that the performances of these advanced models is comparable to standard models (models developed at standard datasets) for N-glycosites’ prediction. This could possibly be explained in conservation of N-glycosite sequon and its strong influence on class divide. However, the predictions of O- and C-glycosites apppeared slightly poor with Advanced GlycoEP models than the performances seen with models trained on standard datasets (Table 3). This can easily be understood in over-optimization of (standard) GlycoEP models for its high similarity and redundancy in overlapping pattern datasets especially for O- and C-glycosites’s standard datasets where as high as 50% of the patterns used in training sets are more similar than 60% (Table 1). Further, decreased size of advanced training datasets post redundancy-reduction at the level of glycosite patterns (almost 50% decrease in the total number of glycosites in standard datasets for O- and C- glycosites, respectively) also contributed in lowering the measured performance of advanced models. However, advanced GlycoEP models are infact more stringently trained on highly non-redundant datasets and therefore, are more robust than standard models in prediction of glycosites. Particularly for the prediction of O- and C- glycosites, advanced models are the most (and probably the only) useful models as advanced datasets employed in training are highly non-redundant therefore, superior to corresponding standard datsets. Further, as advanced GlycoEP models don’t suffer from over-optimization, therefore reports more meaningful. These models have been trained, tested and evaluated using five fold cross validation technique.

**Table 3:** The performance of models developed on advanced datasets for predicting N-linked, O-linked and C-linked glycosites.

Datasets	Type	Sensitivity	Specificity	Accuracy	MCC	AUC
Advanced datasets	N-linked	98.16±0.54	82.82±0.58	84.24±0.49	0.54±0.001	0.93±0.001
	O-linked	35.75±6.28	90.26±0.79	86.87±0.86	0.20±0.05	0.71±0.02
	C-linked	70.67±8.94	93.59±2.98	91.43±3.999	0.78±0.1	0.92±0.08
Advanced datasets (Balanced patterns)	N-linked	98.25±0.53	86.27±1.02	92.26±0.42	0.85±0.01	0.929±0.001
	O-linked	63.4±9.57	62.13±17.31	62.77±5.48	0.27±0.13	0.69±0.08
	C-linked	82.67±16.73	80±36.13	79.82±14.77	0.66±0.22	0.91±0.09

As well as performance on balanced patterns of advanced datasets (results with standard deviation of five fold).

### Performance of Standard Models on Independent Datasets

As discussed in [32], the separation of independent (blind/testing) datasets from training data is an important consideration for better PTM predictions. Therefore, as an additional evaluation measure, we sub-divided entire sequence based non-redundant dataset randomly in to standard datasets (trainset) and corresponding independent datasets (blind-test-set: where none of the proteins in independent dataset was used for training dataset). Details and number of glycosites included in each of these independent datasets are listed in Table 4. Finally we have checked performances of standard models on independent datasets. From the results we found and concluded that the performance of our models is equally well on independent datasets (Table 4).

**Table 4:** The performance of models on an independent datasets, these models were developed on standard datasets.

Types	No. of patterns	Sensitivity	Specificity	Accuracy	MCC	AUC
N-linked	521	96.93	88.87	92.90	0.86	0.935
O-linked	90	72.22	74.44	73.33	0.47	0.783
C-linked	9	100.00	88.89	94.44	0.89	1.000

### Comparison of Standard Models with Existing Methods

As discussed in Introduction section, a good number of computational methods are available for prediction of potential glycosites in eukaryotes. In these, methods like NetOGlyc, EnsembleGly and GPP, have been developed using same glycoprotein datasets as described in previous study, especially for mucin linked O-glycosites prediction [33]. These datasets are not only small but redundant. Our prediction methods have been developed to predict glycosites in similar fashion as in existing approaches, but we have employed larger and a non-redundant datasets that has led to better performances of our models. Among existing methods NetOglyc and NetNglyc are most widely used in prediction of O-linked and N-linked glycosylation, respectively. We have compared prediction results of our best performing SVM models using sequence based non-redundant datasets (standard model) with NetOglyc, NetNglyc, EnsembleGly and Glycosylation Prediction Program (GPP) according to existing comparison approaches. For this comparision purposes we have used the same dataset of 216 glycoproteins as test set that was employed in development of the above-mentioned methods. Standard SVM Models (GlycoEP) developed by us performed better than these programmes and achieved 95.67%, 91.89% and 85.71% accuracy for N-, O- and C-linked glycosite predictions, respectively. Our SVM models could predict Ser and Thr (O-linked glycosylation) with 93.50% and 91.06% accuracy, respectively (Table 5), suggesting an overall better prediction performances of standard GlcoyEP models as well as their applicability to eukaryotic glycosite analysis. In this study, we have compared existing methods with our standard datasets because all existing methods used sequence based non-redundant datasets (standard datasets). Our advanced datasets results are also comparable with these results.

**Table 5:** Comparative performances of existing method with our model developed on standard datasets.

Glycosites	Methods	Sensitivity	Specificity	Accuracy	MCC
**N-linked**	**GlycoEP**	**97.64**	**93.70**	**95.67**	**0.91**
	GPP^1^	96.6	91.8	92.8	0.85
	EnsembleGly^2^	98.0	77.0	95.0	0.84
	NetNglyc^3^	43.9	95.7	76.7	0.49
**O-linked** **(Overall)**	**GlycoEP**	**89.37**	**88.82**	**91.89**	**0.83**
	GPP^1^	94.9	90.7	91.4	0.83
	EnsembleGly^2^	59.0	68.0	89.0	0.64
	NetOglyc^4^	76.0	92.8	88.6	0.66
**O-linked (Ser)**	**GlycoEP**	**87.86**	**92.48**	**93.50**	**0.82**
	GPP^1^	96.1	88.9	90.8	0.81
	NetOglyc^4^	66.7	95.3	91.8	0.62
**O-linked (Thr)**	**GlycoEP**	**86.14**	**95.97**	**91.06**	**0.83**
	GPP^1^	93.6	92.4	92.0	0.84
	NetOglyc^4^	81.5	89.5	84.9	0.67
**C-linked**	GlycoEP	89.80	81.63	85.71	0.72
	EnsembleGly^2^	79.0	77.0	83.0	0.63

Note: GlycoEP -http://www.imtech.res.in/raghava/glycoep/, 1- http://www.comp.chem.nottingham.ac.uk/glyco/, 2- http://www.turing.cs.iastate.edu/EnsembleGly/, 3- http://www.cbs.dtu.dk/services/NetNGlyc/, 4- http://www.cbs.dtu.dk/services/NetOGlyc.

However, as we have discussed already the standard dataset though would qualify as non-redundant dataset at the level of full-length protein sequences, it does carry significant similarity at the overlapping pattern level (Table 1). This may contribute towards certain amount of overprediction in any training method. It would also mean that for a more robust prediction it is desirable to develop methods on a dataset where patterns in training set are highly non-redundant. Therefore, we have developed another set of non-redundant datasets termed advanced datasets as defined already and have developed advanced models using the same.

### N-glycosite Prediction Performance of SVM Using N-linked Sequon as a Feature

In general, all existing glycosites’ prediction algorithms are based on the assumption that either the sequence or structural or both contexts around N-glycosylated Asn (present in the sequon N-X-S/T, where X could be any residue but not proline) are different from the non-glycosylated Asn residues in a given glycoprotein sequence. Therefore, most algorithms including GlycoEP have been trained on the datasets where the positive datasets contain N-X-S/T as positive pattern and negative datasets contain any non glycosylated Asn (including the one which is residing within N-X-S/T sequon but not known to be glycosylated). We, in this study have attempted to exploit the contextual difference around glycosylated sequon (as defined above) versus non-glycosylated sequon datasets composed of sequon containing patterns only. However, our algorithms trained on these datasets could not indicate any further improvement in the prediction results. The purpose of improving class divide might have been defeated here if significant number of yet unidentified (experimentally) but positive glycosites patterns are present in negative datasets. To conclude, the approach where sequons are used only in positive dataset is found better than using it in both positive and negative datasets. The result are presented in Table S17.

## Discussion and Conclusions

For the increased association of glycosylation and phenomenon of pathogenesis/cancer/and other immunity disorders in human [1], [2], [3], [4], [5], the research is growing fast in the field of glycoproteomics. The tools available so far for the prediction of glycosites/residues in a given protein sequence are all based on a small and redundant datasets, highest being a dataset of 216 glycoproteins for the prediction of O-linked glycosites. In this study, we have used the largest and the recent dataset of eukaryotic glycoproteins for the analysis and development of a comprehensive prediction server for eukaryotic glycosites. We have trained SVM classifier and seven WEKA classifiers using balanced datasets and have found SVM performing significantly better (Table S2, S3, S4, S5, S6, S7, S8, S9, S10). The models developed using SVM as a learning tool, could provide significantly enhanced performance for prediction of N-, O- and C- glycosites in eukaryotes. Additionally, we have employed secondary structure and surface accessiblility of residues as an add-on input features in all prediction schemes. Such structural features/context considerations are yet not employed by GPP, EnsembleGly and NetNglyc. The inclusion of ASA indeed, could improve the O-glycosite prediction in general and in all our O-glycosite prediction methods. The overall performance was found better in comparision to the performance of EnsembleGly and NetOglyc. These SVM models provided satisfactory prediction with standard as well advanced datasets. The optimized standard GlycoEP (SVM) models were then compared with four of the best known methods/servers namely, NetNglyc, EnsembleGly, GPP for N-glycosite prediction, NetOglyc, EnsembleGly, GPP for O-glycosite prediction and EnsembleGly for C-glycosite predictions, respectively. The N- and C-glycosite prediction models developed in this study performed better than the all others, wheras O-glycosite prediction was better than the performance of NetOGlyc and EnsembleGly only. However, as expected the redundancy reduction achieved at full-length protein sequence doesn’t result in equivalent redundancy reduction at pattern level (that are used for training the machine tools) implying that the models developed from such data would suffer over-optimization hence over reporting of the performances. Therefore, to address over-optimization and further improve the existing glycosites’ prediction approach we have generated advanced datasets as defined above and have developed advanced glycosite prediction models for N-, O- and C-linked glycosites, respectively. From our results we finally conclude that advanced models are the most strigently trained and more robust than standard models. Further, due to high redundancy in standard datasets of O- and C- glycosites (at pattern level) we understand that advanced model are the only useful models for prediction of O- and C- glycosites and one of the best available for O- and C- glycosites’ prediction. Finally, we have implemented these models at GlycoEP webserver described next. Apart from this, in our N-glycosite dataset of 2604 glycosites we have found exceptions (that we have listed in Table S1) for the universal consensus sequon for N–linked glycosylation (N-X-S/T where X could be any amino acid but not Pro). In order to facilitate easy visualisation of sequons including these rare sequons, we have developed an additional tool named Sequon Scanner. Sequon scanner can scan one or more input sequence for the presence of sequons listed in Table S1 and highlight them in Bold and red colour. In conclusion, in this study we have developed SVM models using patterns based non-redundant datasets for prediction of N-, O- and C-linked glycosylation Further, this study facilitates all three types of prediction at one platform with an option to use standard and/or advanced GlycoEP methods. Additional tool Sequon scanner is provided on the same server.

## Description of Web-Server

The prediction methods described in this paper are implemented in the form of a web-server GlycoEP. The common gateway interface script of GlycoEP is written using CGI/PERL script. This server allows users to predict glycosites in a protein from its amino acid sequence. GlycoEP server allows submission of multiple sequences for prediction. The web server "GlycoEP" is available at http://www.imtech.res.in/raghava/glycoep in an open access mode.

## Supporting Information

Figure S1 **Analysis of Amino acid frequency around consensus sequence for N-glycosites in eukaryotic glycoproteins from** −**10 to +10 sequence length.**
(DOCX)

Figure S2 **Analysis of Amino acid frequency around consensus sequence for O-glycosites (Ser) in eukaryotic glycoproteins from** −**10 to +10 sequence length.**
(DOCX)

Figure S3 **Analysis of Amino acid frequency around consensus sequence for O-glycosites (Thr) in eukaryotic glycoproteins from** −**10 to +10 sequence length.**
(DOCX)

Figure S4 **Spread of glycosylation sites across protein length in eukaryotic N-linked (upper panel) and O-linked (lower panel) glycoproteins.**
(DOCX)

Table S1 **List of exceptional N-linked glycosylation site sequons as retrieved from glycoprotein entries for eukaryotes in Swiss-Prot database (June 2011 release).**
(DOCX)

Table S2 **The performance of Weka classifiers based model developed on standard datasets for predicting N-glycosites using BPP as input feature.**
(DOCX)

Table S3 **The performance of Weka classifiers based model developed on standard datasets for predicting O-glycosites using BPP as input feature.**
(DOCX)

Table S4 **The performance of Weka classifiers based model developed on standard datasets for predicting C-glycosites using BPP as input feature.**
(DOCX)

Table S5 **The performance of Weka classifiers based model developed on standard datasets for predicting N-glycosites using CPP as input feature.**
(DOCX)

Table S6 **The performance of Weka classifiers based model developed on standard datasets for predicting O-glycosites using CPP as input feature.**
(DOCX)

Table S7 **The performance of Weka classifiers based model developed on standard datasets for predicting C-glycosites using CPP as input feature.**
(DOCX)

Table S8 **The performance of Weka classifiers based model developed on standard datasets for predicting N-glycosites using PPP as input feature.**
(DOCX)

Table S9 **The performance of Weka classifiers based model developed on standard datasets for predicting O-glycosites using PPP as input feature.**
(DOCX)

Table S10 **The performance of Weka classifiers based model developed on standard datasets for predicting C-glycosites using PPP as input feature.**
(DOCX)

Table S11 **Performance of SVM using single (CPP or BPP or PPP) or multiple input features (SS and/or ASA) in the prediction of N-linked glycosylation sites using standard datasets.**
(DOCX)

Table S12 **Performance of SVM using single (CPP or BPP or PPP) or multiple input features (with SS and/or ASA) in the prediction of O-linked glycosylation sites using standard datasets.**
(DOCX)

Table S13 **Performance of SVM using single (CPP or BPP or PPP) or multiple input features (SS and/or ASA) in the prediction of C-linked glycosylation sites using standard datasets.**
(DOCX)

Table S14 **Performance of SVM classifier for prediction of eukaryotic N-linked glycosylation sites using BPP/CPP/PPP alone or in combination with SS and ASA as input features on balanced patterns of standard datasets.**
(DOCX)

Table S15 **Performance of SVM classifier for prediction of eukaryotic C-linked glycosylation sites using BPP/CPP/PPP alone or in combination with SS and ASA as input features on balanced patterns of standard datasets.**
(DOCX)

Table S16 **Performance of SVM classifier for prediction of eukaryotic O-linked glycosylation sites using BPP/CPP/PPP alone or in combination with SS and ASA as input features on balanced patterns of standard datasets.**
(DOCX)

Table S17 **Performance of SVM using conserved sequon information along with BPP, CPP or PPP as input features for prediction of N-linked glycosylation sites using Sequon datasets.**
(DOCX)

## References

[1] Hart GW (1992) Glycosylation. Curr Opin Cell Biol 4: 1017–1023. 1485955 10.1016/0955-0674(92)90134-x

[2] Haltiwanger R, Lowe J (2004) Role of Glycosylation in Development. Annual Review of Biochemistry 73: 491–537. 10.1146/annurev.biochem.73.011303.074043 15189151

[3] Miyamoto S (2006) Clinical applications of glycomic approaches for the detection of cancer and other diseases. Curr Opin Mol Ther 8: 507–513. 17243486

[4] Helenius A, Aebi M (2004) Roles of N-linked glycans in the endoplasmic reticulum. Annu Rev Biochem 73: 1019–1049. 15189166 10.1146/annurev.biochem.73.011303.073752

[5] Helenius A, Aebi M (2001) Intracellular Functions of N-linked glycans. Science 291: 2364–2369. 11269317 10.1126/science.291.5512.2364

[6] Gavel Y, von Heijne G (1990) Sequence differences between glycosylated and non-glycosylated Asn-X-Thr/Ser acceptor sites: implications for protein engineering. Protein Eng 3(5): 433–442. 2349213 10.1093/protein/3.5.433 PMC7529082

[7] Lehle L, Tanner W (1978) Glycosyl transfer from dolichyl phosphate sugars to endogenous and exogenous glycoprotein acceptors in yeast. Eur J Biochem 83(2): 563–570. 344041 10.1111/j.1432-1033.1978.tb12124.x

[8] Wilson B, Gavel Y, von Heijne G (1991) Amino acid distributions around O-linked glycosylation sites. Biochem J 275: 529–534. 2025231 10.1042/bj2750529 PMC1150083

[9] Christlet THT, Veluraja K (2001) Database analysis of O-glycosylation sites in proteins. Biophys J 80: 952–960. 11159462 10.1016/s0006-3495(01)76074-2 PMC1301293

[10] Krieg J, Hartmann S, Vicentini A, Glasner W, Hess D, et al (1998) Recognition Signal for C-Mannosylation of Trp-7 in RNase 2 Consists of Sequence Trp-x-x-Trp. Mol Biol Cell 9: 301–309. 9450956 10.1091/mbc.9.2.301 PMC25254

[11] Bhat AH, Mondal H, Chauhan JS, Raghava GP, Methi A, et al.. (2012) ProGlycProt: a repository of experimentally characterized prokaryotic glycoproteins. Nucleic Acids Res: D388–93. 10.1093/nar/gkr911 PMC3245024 22039152

[12] Chauhan JS, Bhat AH, Raghava GP, Rao A (2012) GlycoPP: A Webserver for Prediction of N- and O-Glycosites in Prokaryotic Protein Sequences. PLoS One 7(7): e40155. 22808107 10.1371/journal.pone.0040155 PMC3392279

[13] Dell A, Galadari A, Sastre F, Hitchen P (2010) Similarities and differences in the glycosylation mechanisms in prokaryotes and eukaryotes. Int J Microbiol: 148–178. 10.1155/2010/148178 PMC3068309 21490701

[14] Pandhal J, Wright PC (2010) N-Linked glycoengineering for human therapeutic proteins in bacteria. Biotechnol Lett. 32(9): 1189–98. 10.1007/s10529-010-0289-6 20449632

[15] von der Lieth CW, Bohne-Lang A, Lohmann K, Frank M (2004) Bioinformatics for glycomics: Status, methods, requirements and perspectives. Briefings in Bioinformatics 5(2): 164–178. 15260896 10.1093/bib/5.2.164

[16] Eisenhaber B, Bork P, Eisenhaber F (1999) Prediction of Potential GPI-modification Sites in Protein Sequences. J Mol Biol 292: 741–758. 10497036 10.1006/jmbi.1999.3069

[17] Hansen JE, Lund O, Tolstrup N, Gooley AA, Williams KL, et al (1998) NetOglyc: prediction of mucin type O-glycosylation sites based on sequence context and surface accessibility. Glycoconj J 15: 115–130. 9557871 10.1023/a:1006960004440

[18] Julenius K, Mølgaard A, Gupta R, Brunak S (2005) Prediction, conservation analysis and structural characterization of mammalian mucin-type O-glycosylation sites. Glycobiology 15: 153–164. 15385431 10.1093/glycob/cwh151

[19] Li S, Liu B, Zeng R, Cai Y, Li Y (2006) Predicting O-glycosylation sites in mammalian proteins by using SVMs. Comput Biol Chem 30(3): 203–208. 16731044 10.1016/j.compbiolchem.2006.02.002

[20] Gupta R, Brunk S (2002) Prediction of glycosylation across the human proteome and the correlation to protein function. Pacific Symposium on Biocpmputing 7: 310–322. 11928486

[21] Blom N, Sicheritz-Ponten T, Gupta R, Gammeltoft S, Brunak S (2004) Prediction of post-translational glycosylation and phosphorylation of proteins from the amino acid sequence. Proteomics 4(6): 1633–1649. 15174133 10.1002/pmic.200300771

[22] Caragea C, Sinapov J, Silvescu A, Dobbs D, Honavar V (2007) Glycosylation site prediction using ensembles of Support Vector Machine classifiers. BMC Bioinformatics 8: 438. 17996106 10.1186/1471-2105-8-438 PMC2220009

[23] Hamby SE, Hirst JD (2008) Prediction of glycosylation sites using random forests. BMC Bioinformatics 9: 500. 19038042 10.1186/1471-2105-9-500 PMC2651179

[24] Schwartz D, Chou MF, Church GM (2009) Predicting protein post-translational modifications using meta-analysis of proteome scale data sets. Mol Cell Proteomics. 8(2): 365–79. 10.1074/mcp.M800332-MCP200 PMC2634583 18974045

[25] Hall M, Frank E, Holmes G, Pfahringer B, Reutemann P (2009) The WEKA Data Mining Sogftware: An Update SIGKDD Explorations. 11(1): 10–18.

[26] Vapnik V (1995) The nature of statistical learning theory. New York: Springer.

[27] Joachims T (1999) Making large scale SVM learning practical. Scholkopf B, Burges C, Smola A, editors. Advances in kernel methods: Support Vector Learning. Cambridge: MIT Press 169–184.

[28] Kumar M, Gromiha M, Raghava GPS (2007) Prediction of RNA binding sites in a protein using SVM and PSSM profile. Proteins 71: 189–194. 10.1002/prot.21677 17932917

[29] Chauhan JS, Mishra NK, Raghava GPS (2010) Prediction of GTP interacting residues, dipeptides and tripeptides in a protein from its evolutionary information. BMC Bioinformatics 11: 301. 20525281 10.1186/1471-2105-11-301 PMC3098072

[30] McGuffin LJ, Bryson K, Jones DT (2000) The PSIPRED protein structure prediction server. Bioinformatics 16(4): 404–5. 10869041 10.1093/bioinformatics/16.4.404

[31] Garg A, Kaur H, Raghava GPS (2005) Real value prediction of solvent accessibility in proteins using multiple sequence alignment and secondary structure. Proteins 61(2): 318–324. 16106377 10.1002/prot.20630

[32] Schwartz D (2012) Prediction of lysine post-translational modifications using bioinformatic tools. Essays Biochem 52: 165–77. 22708570 10.1042/bse0520165

[33] Gupta R, Birch H, Rapacki K, Brunak S, Hansen J (1999) O-GLYCBASE version 4.0: a revised database of O-glycosylated proteins. Nucleic Acids Res 27: 370–372. 9847232 10.1093/nar/27.1.370 PMC148187

