# Supplementary material for: In silico Platform for Prediction of N-, O- and C-Glycosites in Eukaryotic Protein Sequences
Source: PLoS One. 2013 Jun 28;8(6):e67008. doi: 10.1371/journal.pone.0067008 (PMC3695939; doi:10.1371/journal.pone.0067008)

**Figure: S3**. Analysis of Amino acid frequency around consensus sequence for O-linked Glycosylation (Thr) in eukaryotic glycoproteins from -10 to + 10 sequence length.


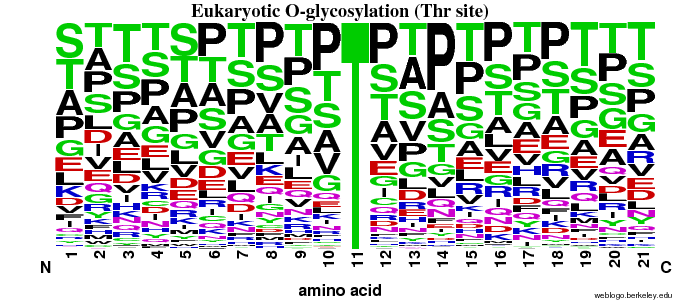

Supplement: Figure S3 — Analysis of Amino acid frequency around consensus sequence for O-glycosites (Thr) in eukaryotic glycoproteins from −10 to +10 sequence length. (DOCX) [file pone.0067008.s003.docx]
