# Supplementary material for: In silico Platform for Prediction of N-, O- and C-Glycosites in Eukaryotic Protein Sequences
Source: PLoS One. 2013 Jun 28;8(6):e67008. doi: 10.1371/journal.pone.0067008 (PMC3695939; doi:10.1371/journal.pone.0067008)

**Figure S4:** Spread of glycosylation sites across protein length in eukaryotic N-linked (upper panel) and O-linked (lower panel) glycoproteins.


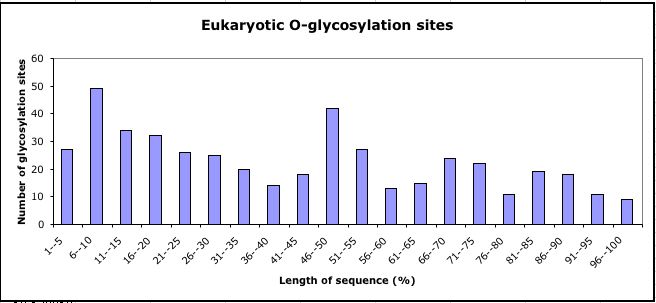

Supplement: Figure S4 — Spread of glycosylation sites across protein length in eukaryotic N-linked (upper panel) and O-linked (lower panel) glycoproteins. (DOCX) [file pone.0067008.s004.docx]
