# Supplementary material for: In silico Platform for Prediction of N-, O- and C-Glycosites in Eukaryotic Protein Sequences
Source: PLoS One. 2013 Jun 28;8(6):e67008. doi: 10.1371/journal.pone.0067008 (PMC3695939; doi:10.1371/journal.pone.0067008)
Supplement: Table S1 — List of exceptional N-linked glycosylation site sequons as retrieved from glycoprotein entries for eukaryotes in Swiss-Prot database (June 2011 release). (DOCX) [file pone.0067008.s005.docx]

**Table S1:** List of exceptional N-linked glycosylation site sequons as retrieved from glycoprotein entries for eukaryotes in Swiss-Prot database (June 2011 release).

| **N---X---C Tripeptide Consensus Sequence** | | | | | | |
| --- | --- | --- | --- | --- | --- | --- |
| S. No. | Proteins Ids with N-glyco site position | | Organisms | | Cellular Components | 21 AA length pattern Sequence |
| 1 | O08543 [162] | | Mus musculus | | Cell membrane | LKLKVFVRPTNSCMKTIGVHD |
| 2 | P08575 [284] | | Homo sapiens | | Membrane | CKNASVSISHNSCTAPDKTLI |
| 3 | P34576 [3337] | | C. elegans | | Cell membrane | RDVDECALGLNNCSGVAHCID |
| 4 | P43510 [201] | | C. elegans | | Not mentioned | IVTGSNYTANNGCKPYPFPPC |
| 5 | Q04457 [73] | | C. elegans | | E.R. | EDVYPATQYRNDCTPHYRLVA |
| 6 | Q09163 [174] | | Mus musculus | | Membrane | GNFCEIVAATNSCTPNPCEND |
| 7 | Q19981 [832] | | C. elegans | | Membrane | TECPMPCAQRNNCSDCTDLEQ |
| 8 | Q24114 [97] | | Drosophila | | Cell membrane | YSEKGAICGGNCCNNATELEL |
| 9 | Q8R2Q8 [94] | | Mus musculus | | Cell membrane | DSLLQAETQANSCNLTVVTLQ |
| **N---X---L Tripeptide Consensus Sequence** | | | | | | |
| 1 | P11688 [675] | | Mus musculus | | Membrane | EKKHVYLGDK**N**ALNLTFHAQN |
| 2 | P19137 [2062] | | Mus musculus | | Membrane | SRVNATVQET**N**DLLHNSTMTT |
| 3 | P20241 [414] | | Drosophila | | Cell junction | DTGNYGCNAT**N**SLGYVYKDVY |
| 4 | Q63HQ0 [256] | | Homo sapiens | | Endosome | SNPTSASDDS**N**GLEWENDFVS |
| 5 | Q6GU68 [60] | | Mus musculus | | Secreted | ANVTTLSLSA**N**RLPGLPEGAF |
| 6 | Q8C0Z1 [119] | | Mus musculus | | Membrane | LFLYKNTNSS**N**NLTRSCADEG |
| 7 | Q8R373 [72] | | Mus musculus | | Cell junction | VITYSSRHVY**N**NLTEEQKGRV |
| 8 | Q8R5M8 [304] | | Mus musculus | | Cell membrane | AVLSGPNLFI**N**NLNKTDNGTY |
| 9 | Q92854 [74] | | Homo sapiens | | Membrane | IGAREAVFAV**N**ALNISEKQHE |
| 10 | Q96DU3 [171] | | Homo sapiens | | Cell membrane | NVSFRWEALG**N**TLSSQPNLTV |
| 11 | Q9ER38 [109] | | Mus musculus | | Cytoplasm | CSGGGDCRIS**N**NLTGLESDLR |
| **N---X---Q Tripeptide Consensus Sequence** | | | | | | |
| 1 | P10586 [959] | | Homo sapiens | | Membrane | ISYTVVFRDI**N**SQQELQNITT |
| 2 | P10674 [493] | | Drosophila | | Cell membrane | TYKMGKFSHF**N**DQLNNTQRRF |
| 3 | P11276 [1001] | | Mus musculus | | Extracellular matrix | QQTTKLDAPT**N**LQFVNETDRT |
| 4 | P18572 [309] | | Mus musculus | | Cell membrane | DPGTYVCNAT**N**AQGTTRETIS |
| 5 | P30825 [216] | | Homo sapiens | | Membrane | VSGFVKGSVK**N**WQLTEEDFGN |
| 6 | Q6ZUK4 [116] | | Homo sapiens | | Membrane | KEDFNQTLTS**N**EQTSRADDLI |
| **N---X---N Tripeptide Consensus Sequence** | | | | | | |
| 1 | O09117 [70] | | Mus musculus | | Cytoplasmic vesicle | IQVNCPKVGVNKNQTVTATFG |
| 2 | P13598 [103] | | Homo sapiens | | Membrane | FTCSGKQESMNSNVSVYQPPR |
| 3 | P55012 [544] | | Mus musculus | | Membrane | GSCVVRDATGNVNDTITTELT |
| 4 | Q6P9J9 [492] | | Mus musculus | | Ion transport | FIVFSTTLPKNPNGTDPIQKY |
| 5 | Q8C0Z1 [114] | | Mus musculus | | Membrane | KVQDVLFLYKNTNSSNNLTRS |
| 6 | Q9VN14 [633] | | Drosophila | | Cell junction | RVLIIQNATTNDNGEYSCTIT |
| **N---X---E Tripeptide Consensus Sequence** | | | | | | |
| 1 | P08575 [240] | | Homo sapiens | | Membrane | YANITVDYLYNKETKLFTAKL |
| 2 | P09208 [1219] | | Drosophila | | Membrane | DLKVDLEHANNTESPVRVRWT |
| 3 | Q3TWN3 [111] | | Mus musculus | | Ion transport | VKLRVYGQNINNETWSRIAFT |
| 4 | Q9BX67 [198] | | Homo sapiens | | Cell membrane | PRFRNSSFHLNSETGTLVFTA |
| 5 | Q9Z0M6 [407] | | Mus musculus | | Cell membrane | SSVAGILSSPNMEKLLGNTPL |
| **N---X---R Tripeptide Consensus Sequence** | | | | | | |
| 1 | A2ARV4 [399] | | Mus musculus | | Coated pit | SFSAASIIFSNGRDLLVGDLH |
| 2 | P32942 [91] | | Homo sapiens | | Membrane | AAFNLSNVTGNSRILCSVYCN |
| 3 | Q01151 [86] | | Homo sapiens | | Membrane | KGQNGSFDAPNERPYSLKIRN |
| 4 | Q13740 [466] | | Homo sapiens | | Membrane | INQTEESPYINGRYYSKIIIS |
| **N---X---K Tripeptide Consensus Sequence** | | | | | | |
| 1 | P13595 [453] | | Mus musculus | | Cell membrane | GQLLPSSNYSNIKIYNTPSAS |
| 2 | P18572 [275] | | Mus musculus | | Cell membrane | EEAITNSTEANGKYVVVSTPE |
| 3 | P21995 [210] | | Mus musculus | | Membrane | TAQVPIDAHSNEKYIINGSHA |
| 4 | Q5FWI3 [282] | | Mus musculus | | Membrane | IDQDTARVLENEKFDTHEYHN |
| **N---X---I Tripeptide Consensus Sequence** | | | | | | |
| 1 | P21995 [76] | | Mus musculus | | Membrane | TEKSNVSVEENVILEKPSHVE |
| 2 | Q91VA1[201] | | Mus musculus | | Membrane | DLRINNTTVSNGISGLLDSIN |
| 3 | Q9W568 [268] | | Drosophila | | Not mention | SALKCLNISNNNISEIHSRAV |
| **N---X---F Tripeptide Consensus Sequence** | | | | | | |
| 1 | Q63HQ0 [262] | | Homo sapiens | | Endosome | SDDSNGLEWENDFVSAEMDDN |
| 2 | Q8K4Q8 [160] | | Mus musculus | | Membrane | QSQLKETLQNNSFLITTVNKT |
| 3 | Q99523 [163] | | Homo sapiens | | Cell membrane | NFKDITDLINNTFIRTEFGMA |
| **Others Tripeptide Consensus Sequences** | | | | | | |
| N---X---V | | P09326 [178] | | Homo sapiens | Cell membrane | DKRPFPKELQNSVLETTLMPH |
| N---X---V | | P56564 [215] | | Mus musculus | Membrane | SNETLLGAVINNVSEAMETLT |
| N---X---A | | P01848 [61] | | Homo sapiens | Membrane | LDMRSMDFKSNSAVAWSNKSD |
| N---X---A | | Q62470 [599] | | Mus musculus | Membrane | LRSLDAYPVLNQAQAMENHTE |
| N---X---D | | P08195 [395] | | Homo sapiens | Cell membrane | LQQILSLLESNKDLLLTSSYL |
| N---X---D | | P14094 [163] | | Mus musculus | Membrane | LDWLGNCSGLNDDSYGYREGK |
| N---X---M | | P08575 [497] | | Homo sapiens | Membrane | WNMTVSMTSDNSMHVKCRPPR |
| N---X---M | | P21995 [98] | | Mus musculus | Membrane | KCVYTATKDLNLMNVTWKKDD |
| N---X---W | | P08195 [428] | | Homo sapiens | Cell membrane | LVTQYLNATGNRWCSWSLSQA |
| N---X---P | | Q99MR3 [218] | | Mus musculus | Cell membrane | LVSFVAVGPRNIPLAPRPGTN |
| N---X---Y | | P05622 [312] | | Mus musculus | Membrane | EKAINISVIENGYVRLLETLG |
| N---X---G | | P11688 [685] | | Mus musculus | Membrane | NALNLTFHAQNLGEGGAYEAE |
