# Supplementary material for: In silico Platform for Prediction of N-, O- and C-Glycosites in Eukaryotic Protein Sequences
Source: PLoS One. 2013 Jun 28;8(6):e67008. doi: 10.1371/journal.pone.0067008 (PMC3695939; doi:10.1371/journal.pone.0067008)
Supplement: Table S2 — The performance of Weka classifiers based model developed on standard datasets for predicting N-glycosites using BPP as input feature. (DOCX) [file pone.0067008.s006.docx]

**Table S2**: The performance of Weka classifiers based model developed on standard datasets for predicting N-glycosites using BPP as input feature.

| Clasifier | Precision | Recall | F-Measure | AUC | ACC |
| --- | --- | --- | --- | --- | --- |
| SVM**^light^** | 0.878 | 0.975 | 0.923 | 0.928 | 92.05 |
| LibSVM | 0.926 | 0.921 | 0.92 | 0.921 | 92.05 |
| RBFNetwork | 0.895 | 0.895 | 0.895 | 0.906 | 89.47 |
| SMO | 0.925 | 0.921 | 0.921 | 0.921 | 92.10 |
| LMT | 0.925 | 0.921 | 0.92 | 0.926 | 91.00 |
| RandomForest | 0.882 | 0.878 | 0.878 | 0.915 | 87.80 |
| BayesNet | 0.926 | 0.922 | 0.922 | 0.93 | 92.20 |
| NaiveBayes | 0.851 | 0.851 | 0.851 | 0.908 | 85.10 |
