# Supplementary material for: In silico Platform for Prediction of N-, O- and C-Glycosites in Eukaryotic Protein Sequences
Source: PLoS One. 2013 Jun 28;8(6):e67008. doi: 10.1371/journal.pone.0067008 (PMC3695939; doi:10.1371/journal.pone.0067008)
Supplement: Table S3 — The performance of Weka classifiers based model developed on standard datasets for predicting O-glycosites using BPP as input feature. (DOCX) [file pone.0067008.s007.docx]

**Table S3**: The performance of Weka classifiers based model developed on standard datasets for predicting O-glycosites using BPP as input feature.

| Clasifier | Precision | Recall | F-Measure | AUC | ACC |
| --- | --- | --- | --- | --- | --- |
| SVM**^light^** | 0.693 | 0.747 | 0.718 | 0.772 | 70.84 |
| LibSVM | 0.684 | 0.667 | 0.66 | 0.667 | 66.74 |
| RBFNetwork | 0.629 | 0.629 | 0.629 | 0.643 | 62.86 |
| SMO | 0.686 | 0.684 | 0.683 | 0.684 | 68.40 |
| LMT | 0.661 | 0.661 | 0.661 | 0.712 | 66.07 |
| RandomForest | 0.631 | 0.623 | 0.617 | 0.661 | 62.30 |
| BayesNet | 0.594 | 0.593 | 0.593 | 0.634 | 59.31 |
| NaiveBayes | 0.698 | 0.652 | 0.63 | 0.746 | 65.18 |
