# Supplementary material for: In silico Platform for Prediction of N-, O- and C-Glycosites in Eukaryotic Protein Sequences
Source: PLoS One. 2013 Jun 28;8(6):e67008. doi: 10.1371/journal.pone.0067008 (PMC3695939; doi:10.1371/journal.pone.0067008)
Supplement: Table S4 — The performance of Weka classifiers based model developed on standard datasets for predicting C-glycosites using BPP as input feature. (DOCX) [file pone.0067008.s008.docx]

**Table S4**: The performance of Weka classifiers based model developed on standard datasets for predicting C-glycosites using BPP as input feature.

| Clasifier | Precision | Recall | F-Measure | AUC | ACC |
| --- | --- | --- | --- | --- | --- |
| SVM**^light^** | 0.955 | 0.895 | 0.924 | 0.941 | 92.71 |
| LibSVM | 0.911 | 0.906 | 0.906 | 0.906 | 90.62 |
| RBFNetwork | 0.645 | 0.625 | 0.611 | 0.607 | 62.50 |
| SMO | 0.876 | 0.875 | 0.875 | 0.875 | 87.50 |
| LMT | 0.876 | 0.875 | 0.875 | 0.919 | 87.50 |
| RandomForest | 0.77 | 0.76 | 0.758 | 0.862 | 76.04 |
| BayesNet | 0.813 | 0.813 | 0.812 | 0.859 | 81.25 |
| NaiveBayes | 0.845 | 0.844 | 0.844 | 0.895 | 84.37 |
