# Supplementary material for: In silico Platform for Prediction of N-, O- and C-Glycosites in Eukaryotic Protein Sequences
Source: PLoS One. 2013 Jun 28;8(6):e67008. doi: 10.1371/journal.pone.0067008 (PMC3695939; doi:10.1371/journal.pone.0067008)
Supplement: Table S5 — The performance of Weka classifiers based model developed on standard datasets for predicting N-glycosites using CPP as input feature. (DOCX) [file pone.0067008.s009.docx]

**Table S5**: The performance of Weka classifiers based model developed on standard datasets for predicting N-glycosites using CPP as input feature.

| Clasifier | Precision | Recall | F-Measure | AUC | ACC |
| --- | --- | --- | --- | --- | --- |
| SVM**^light^** | 0.621 | 0.649 | 0.634 | 0.673 | 62.65 |
| LibSVM | 0.56 | 0.534 | 0.475 | 0.534 | 53.36 |
| RBFNetwork | 0.59 | 0.589 | 0.588 | 0.617 | 58.92 |
| SMO | 0.616 | 0.615 | 0.614 | 0.615 | 61.50 |
| LMT | 0.609 | 0.609 | 0.609 | 0.653 | 60.88 |
| RandomForest | 0.585 | 0.581 | 0.576 | 0.613 | 58.08 |
| BayesNet | 0.601 | 0.597 | 0.592 | 0.63 | 59.65 |
| NaiveBayes | 0.604 | 0.591 | 0.577 | 0.64 | 59.06 |
