# Supplementary material for: In silico Platform for Prediction of N-, O- and C-Glycosites in Eukaryotic Protein Sequences
Source: PLoS One. 2013 Jun 28;8(6):e67008. doi: 10.1371/journal.pone.0067008 (PMC3695939; doi:10.1371/journal.pone.0067008)
Supplement: Table S6 — The performance of Weka classifiers based model developed on standard datasets for predicting O-glycosites using CPP as input feature. (DOCX) [file pone.0067008.s010.docx]

**Table S6**: The performance of Weka classifiers based model developed on standard datasets for predicting O-glycosites using CPP as input feature.

| Clasifier | Precision | Recall | F-Measure | ROC-Area | ACC |
| --- | --- | --- | --- | --- | --- |
| SVM**^light^** | 0.733 | 0.707 | 0.719 | 0.786 | 72.28 |
| LibSVM | 0.77 | 0.657 | 0.618 | 0.657 | 65.74 |
| RBFNetwork | 0.686 | 0.685 | 0.685 | 0.718 | 68.51 |
| SMO | 0.75 | 0.746 | 0.745 | 0.746 | 74.61 |
| LMT | 0.694 | 0.694 | 0.694 | 0.725 | 69.40 |
| RandomForest | 0.712 | 0.711 | 0.71 | 0.788 | 71.06 |
| BayesNet | 0.673 | 0.673 | 0.673 | 0.726 | 67.29 |
| NaiveBayes | 0.683 | 0.681 | 0.68 | 0.726 | 68.07 |
