# Supplementary material for: In silico Platform for Prediction of N-, O- and C-Glycosites in Eukaryotic Protein Sequences
Source: PLoS One. 2013 Jun 28;8(6):e67008. doi: 10.1371/journal.pone.0067008 (PMC3695939; doi:10.1371/journal.pone.0067008)
Supplement: Table S7 — The performance of Weka classifiers based model developed on standard datasets for predicting C-glycosites using CPP as input feature. (DOCX) [file pone.0067008.s011.docx]

**Table S7**: The performance of Weka classifiers based model developed on standard datasets for predicting C-glycosites using CPP as input feature.

| Clasifier | Precision | Recall | F-Measure | AUC | ACC |
| --- | --- | --- | --- | --- | --- |
| SVM**^light^** | 0.866 | 0.812 | 0.838 | 0.876 | 84.38 |
| LibSVM | 0.604 | 0.604 | 0.604 | 0.604 | 60.41 |
| RBFNetwork | 0.796 | 0.792 | 0.791 | 0.811 | 79.16 |
| SMO | 0.854 | 0.854 | 0.854 | 0.854 | 85.41 |
| LMT | 0.791 | 0.781 | 0.779 | 0.863 | 78.12 |
| RandomForest | 0.876 | 0.875 | 0.875 | 0.873 | 87.50 |
| BayesNet | 0.836 | 0.833 | 0.833 | 0.857 | 83.33 |
| NaiveBayes | 0.845 | 0.844 | 0.844 | 0.893 | 84.37 |
