# Supplementary material for: In silico Platform for Prediction of N-, O- and C-Glycosites in Eukaryotic Protein Sequences
Source: PLoS One. 2013 Jun 28;8(6):e67008. doi: 10.1371/journal.pone.0067008 (PMC3695939; doi:10.1371/journal.pone.0067008)
Supplement: Table S8 — The performance of Weka classifiers based model developed on standard datasets for predicting N-glycosites using PPP as input feature. (DOCX) [file pone.0067008.s012.docx]

**Table S8:** The performance of Weka classifiers based model developed on standard datasets for predicting N-glycosites using PPP as input feature.

| Classifier | Precision | Recall | F-Measure | AUC | ACC |
| --- | --- | --- | --- | --- | --- |
| SVM**^light^** | 0.7608 | 0.790 | 0.774 | 0.84 | 77.10 |
| LibSVM | 0.781 | 0.772 | 0.77 | 0.772 | 77.15 |
| RBFNetwork | 0.687 | 0.686 | 0.686 | 0.734 | 68.63 |
| SMO | 0.756 | 0.754 | 0.754 | 0.754 | 75.44 |
| LMT | 0.767 | 0.763 | 0.762 | 0.821 | 76.29 |
| RandomForest | 0.679 | 0.673 | 0.671 | 0.729 | 67.32 |
| BayesNet | 0.743 | 0.743 | 0.742 | 0.808 | 74.25 |
| NaiveBayes | 0.715 | 0.715 | 0.715 | 0.766 | 71.49 |
