# Supplementary material for: In silico Platform for Prediction of N-, O- and C-Glycosites in Eukaryotic Protein Sequences
Source: PLoS One. 2013 Jun 28;8(6):e67008. doi: 10.1371/journal.pone.0067008 (PMC3695939; doi:10.1371/journal.pone.0067008)
Supplement: Table S9 — The performance of Weka classifiers based model developed on standard datasets for predicting O-glycosites using PPP as input feature. (DOCX) [file pone.0067008.s013.docx]

**Table S9:** The performance of Weka classifiers based model developed on standard datasets for predicting O-glycosites using PPP as input feature.

| Classifier | Precision | Recall | F-Measure | AUC | ACC |
| --- | --- | --- | --- | --- | --- |
| SVM**^light^** | 0.674 | 0.634 | 0.653 | 0.735 | 67.74 |
| LibSVM | 0.655 | 0.653 | 0.652 | 0.653 | 65.29 |
| RBFNetwork | 0.661 | 0.661 | 0.66 | 0.669 | 66.07 |
| SMO | 0.679 | 0.678 | 0.678 | 0.678 | 67.84 |
| LMT | 0.632 | 0.632 | 0.632 | 0.676 | 63.19 |
| RandomForest | 0.608 | 0.602 | 0.597 | 0.636 | 60.19 |
| BayesNet | 0.593 | 0.592 | 0.59 | 0.646 | 59.20 |
| NaiveBayes | 0.661 | 0.661 | 0.661 | 0.714 | 66.07 |
