# Supplementary material for: In silico Platform for Prediction of N-, O- and C-Glycosites in Eukaryotic Protein Sequences
Source: PLoS One. 2013 Jun 28;8(6):e67008. doi: 10.1371/journal.pone.0067008 (PMC3695939; doi:10.1371/journal.pone.0067008)
Supplement: Table S10 — The performance of Weka classifiers based model developed on standard datasets for predicting C-glycosites using PPP as input feature. (DOCX) [file pone.0067008.s014.docx]

**Table S10:** The performance of Weka classifiers based model developed on standard datasets for predicting C-glycosites using PPP as input feature.

| Clasifier | Precision | Recall | F-Measure | AUC | ACC |
| --- | --- | --- | --- | --- | --- |
| SVM**^light^** | 0.891 | 0.893 | 0.892 | 0.920 | 89.36 |
| LibSVM | 0.887 | 0.883 | 0.883 | 0.883 | 88.29 |
| RBFNetwork | 0.801 | 0.787 | 0.785 | 0.773 | 78.72 |
| SMO | 0.873 | 0.872 | 0.872 | 0.872 | 87.23 |
| LMT | 0.761 | 0.755 | 0.754 | 0.834 | 75.53 |
| RandomForest | 0.857 | 0.851 | 0.85 | 0.926 | 85.10 |
| BayesNet | 0.904 | 0.904 | 0.904 | 0.935 | 90.42 |
| NaiveBayes | 0.845 | 0.83 | 0.828 | 0.842 | 82.97 |
