# Supplementary material for: In silico Platform for Prediction of N-, O- and C-Glycosites in Eukaryotic Protein Sequences
Source: PLoS One. 2013 Jun 28;8(6):e67008. doi: 10.1371/journal.pone.0067008 (PMC3695939; doi:10.1371/journal.pone.0067008)
Supplement: Table S11 — Performance of SVM using single (CPP or BPP or PPP) or multiple input features (SS and/or ASA) in the prediction of N-linked glycosylation sites using standard datasets. (DOCX) [file pone.0067008.s015.docx]

**Table S11:** Performance of SVM using single (CPP or BPP or PPP) or multiple input features (SS and/or ASA) in the prediction of N-linked glycosylation sites using standard datasets.

| Feature | Sensitivity | Specificity | Accuracy | MCC |
| --- | --- | --- | --- | --- |
| CPP | 54.72 | 60.53 | 60.15 | 0.08 |
| CPP +SS | 92.68 | 83.29 | 83.94 | 0.47 |
| CPP +ASA | 89.46 | 84.23 | 83.57 | 0.45 |
| CPP +SS+ASA | 84.78 | 81.67 | 81.88 | 0.40 |
| BPP | 95.97 | 87.38 | 87.95 | 0.54 |
| BPP +SS | 93.39 | 87.81 | 88.19 | 0.54 |
| BPP +ASA | 96.46 | 87.21 | 87.85 | 0.55 |
| BPP +SS+ASA | 93.90 | 88.06 | 88.46 | 0.55 |
| PPP | 73.66 | 76.37 | 76.18 | 0.28 |
| PPP +SS | 69.94 | 77.90 | 77.35 | 0.28 |
| PPP +ASA | 72.42 | 78.27 | 77.87 | 0.30 |
| PPP +SS+ASA | 75.32 | 77.50 | 78.06 | 0.30 |
