# Supplementary material for: In silico Platform for Prediction of N-, O- and C-Glycosites in Eukaryotic Protein Sequences
Source: PLoS One. 2013 Jun 28;8(6):e67008. doi: 10.1371/journal.pone.0067008 (PMC3695939; doi:10.1371/journal.pone.0067008)
Supplement: Table S12 — Performance of SVM using single (CPP or BPP or PPP) or multiple input features (with SS and/or ASA) in the prediction of O-linked glycosylation sites using standard datasets. (DOCX) [file pone.0067008.s016.docx]

**Table S12:** Performance of SVM using single (CPP or BPP or PPP) or multiple input features (with SS and/or ASA) in the prediction of O-linked glycosylation sites using standard datasets.

| Feature | Sensitivity | Specificity | Accuracy | MCC |
| --- | --- | --- | --- | --- |
| CPP | 75.66 | 84.47 | 84.08 | 0.32 |
| CPP +SS | 91.11 | 85.06 | 85.34 | 0.41 |
| CPP +ASA | 98.67 | 84.71 | 85.35 | 0.44 |
| CPP +SS+ASA | 81.11 | 93.70 | 93.12 | 0.53 |
| BPP | 69.62 | 68.17 | 68.23 | 0.16 |
| BPP+SS | 65.78 | 71.30 | 71.05 | 0.17 |
| BPP+ASA | 66.22 | 72.19 | 71.92 | 0.18 |
| BPP+SS+ASA | 68.89 | 72.65 | 72.47 | 0.19 |
| PPP | 73.66 | 76.37 | 76.18 | 0.28 |
| PPP+SS | 64.89 | 73.14 | 72.76 | 0.18 |
| PPP+ASA | 65.33 | 74.65 | 74.22 | 0.19 |
| PPP+SS+ASA | 61.33 | 77.03 | 76.30 | 0.19 |
