# Supplementary material for: In silico Platform for Prediction of N-, O- and C-Glycosites in Eukaryotic Protein Sequences
Source: PLoS One. 2013 Jun 28;8(6):e67008. doi: 10.1371/journal.pone.0067008 (PMC3695939; doi:10.1371/journal.pone.0067008)
Supplement: Table S13 — Performance of SVM using single (CPP or BPP or PPP) or multiple input features (SS and/or ASA) in the prediction of C-linked glycosylation sites using standard datasets. (DOCX) [file pone.0067008.s017.docx]

**Table S13:** Performance of SVM using single (CPP or BPP or PPP) or multiple input features (SS and/or ASA) in the prediction of C-linked glycosylation sites using standard datasets.

| Feature | Sensitivity | Specificity | Accuracy | MCC |
| --- | --- | --- | --- | --- |
| CPP | 85.42 | 86.24 | 85.99 | 0.69 |
| CPP+SS | 83.33 | 86.14 | 85.23 | 0.68 |
| CPP+ASA | 89.58 | 87.13 | 87.92 | 0.74 |
| CPP+SS+ASA | 85.42 | 87.13 | 86.58 | 0.71 |
| BPP | 87.50 | 92.66 | 91.08 | 0.79 |
| BPP+SS | 89.58 | 90.10 | 89.93 | 0.78 |
| BPP+ASA | 91.67 | 90.10 | 90.60 | 0.79 |
| BPP+SS+ASA | 87.50 | 92.08 | 90.60 | 0.79 |
| PPP | 87.50 | 91.74 | 90.45 | 0.78 |
| PPP+SS | 91.67 | 87.13 | 88.59 | 0.76 |
| PPP+ASA | 89.58 | 89.11 | 89.26 | 0.76 |
| PPP+SS+ASA | 89.58 | 90.10 | 89.93 | 0.78 |
