# Supplementary material for: In silico Platform for Prediction of N-, O- and C-Glycosites in Eukaryotic Protein Sequences
Source: PLoS One. 2013 Jun 28;8(6):e67008. doi: 10.1371/journal.pone.0067008 (PMC3695939; doi:10.1371/journal.pone.0067008)
Supplement: Table S14 — Performance of SVM classifier for prediction of eukaryotic N-linked glycosylation sites using BPP/CPP/PPP alone or in combination with SS and ASA as input features on balanced patterns of standard datasets. (DOCX) [file pone.0067008.s018.docx]

**Table S14:** Performance of SVM classifier for prediction of eukaryotic N-linked glycosylation sites using BPP/CPP/PPP alone or in combination with SS and ASA as input features on balanced patterns of standard datasets.

| Feature | Sensitivity | Specificity | Accuracy | MCC | AUC |
| --- | --- | --- | --- | --- | --- |
| CPP | 64.94 | 60.37 | 62.65 | 0.25 | 0.673 |
| CPP+SS | 63.38 | 61.57 | 62.47 | 0.25 | 0.67 |
| CPP+ASA | 67.94 | 63.81 | 65.87 | 0.32 | 0.71 |
| CPP+SS+ASA | 68.17 | 61.41 | 64.79 | 0.30 | 0.695 |
| BPP | 97.58 | 86.52 | 92.05 | 0.85 | 0.928 |
| BPP+SS | 96.70 | 86.70 | 91.70 | 0.84 | 0.927 |
| BPP+ASA | 97.29 | 86.82 | 92.05 | 0.85 | 0.929 |
| BPP+SS+ASA | 97.44 | 86.19 | 91.82 | 0.84 | 0.928 |
| PPP | 79.03 | 75.16 | 77.10 | 0.54 | 0.84 |
| PPP+SS | 78.21 | 76.99 | 77.60 | 0.55 | 0.846 |
| PPP+ASA | 81.00 | 74.47 | 77.73 | 0.56 | 0.842 |
| PPP+SS+ASA | 79.39 | 75.73 | 77.56 | 0.55 | 0.843 |
