# Supplementary material for: In silico Platform for Prediction of N-, O- and C-Glycosites in Eukaryotic Protein Sequences
Source: PLoS One. 2013 Jun 28;8(6):e67008. doi: 10.1371/journal.pone.0067008 (PMC3695939; doi:10.1371/journal.pone.0067008)
Supplement: Table S15 — Performance of SVM classifier for prediction of eukaryotic C-linked glycosylation sites using BPP/CPP/PPP alone or in combination with SS and ASA as input features on balanced patterns of standard datasets. (DOCX) [file pone.0067008.s019.docx]

**Table S15:** Performance of SVM classifier for prediction of eukaryotic C-linked glycosylation sites using BPP/CPP/PPP alone or in combination with SS and ASA as input features on balanced patterns of standard datasets.

| Feature | Sensitivity | Specificity | Accuracy | MCC | AUC |
| --- | --- | --- | --- | --- | --- |
| CPP | 81.25 | 87.50 | 84.38 | 0.69 | 0.876 |
| CPP+SS | 85.42 | 83.33 | 84.38 | 0.69 | 0.840 |
| CPP+ASA | 87.50 | 91.67 | 89.58 | 0.79 | 0.911 |
| CPP+SS+ASA | 85.42 | 91.67 | 88.54 | 0.77 | 0.894 |
| BPP | 89.58 | 95.83 | 92.71 | 0.86 | 0.941 |
| BPP+SS | 87.50 | 95.83 | 91.67 | 0.84 | 0.931 |
| BPP+ASA | 87.50 | 95.83 | 91.67 | 0.84 | 0.946 |
| BPP+SS+ASA | 89.58 | 95.83 | 92.71 | 0.86 | 0.942 |
| PPP | 89.36 | 89.36 | 89.36 | 0.79 | 0.920 |
| PPP+SS | 87.50 | 95.83 | 91.67 | 0.84 | 0.924 |
| PPP+ASA | 89.58 | 95.83 | 92.71 | 0.86 | 0.922 |
| PPP+SS+ASA | 87.50 | 95.83 | 91.67 | 0.84 | 0.924 |
