# Supplementary material for: In silico Platform for Prediction of N-, O- and C-Glycosites in Eukaryotic Protein Sequences
Source: PLoS One. 2013 Jun 28;8(6):e67008. doi: 10.1371/journal.pone.0067008 (PMC3695939; doi:10.1371/journal.pone.0067008)
Supplement: Table S16 — Performance of SVM classifier for prediction of eukaryotic O-linked glycosylation sites using BPP/CPP/PPP alone or in combination with SS and ASA as input features on balanced patterns of standard datasets. (DOCX) [file pone.0067008.s020.docx]

**Table S16:** Performance of SVM classifier for prediction of eukaryotic O-linked glycosylation sites using BPP/CPP/PPP alone or in combination with SS and ASA as input features on balanced patterns of standard datasets.

| Feature | Sensitivity | Specificity | Accuracy | MCC | AUC |
| --- | --- | --- | --- | --- | --- |
| CPP | 70.07 | 74.50 | 72.28 | 0.45 | 0.786 |
| CPP+SS | 77.11 | 80.67 | 78.89 | 0.58 | 0.864 |
| CPP+ASA | 88.22 | 83.78 | 86.00 | 0.72 | 0.941 |
| CPP+SS+ASA | 79.33 | 80.67 | 80.00 | 0.60 | 0.872 |
| BPP | 74.72 | 66.96 | 70.84 | 0.42 | 0.772 |
| BPP+SS | 69.78 | 71.78 | 70.78 | 0.42 | 0.768 |
| BPP+ASA | 74.89 | 69.33 | 72.11 | 0.44 | 0.786 |
| BPP+SS+ASA | 71.78 | 69.78 | 70.78 | 0.42 | 0.769 |
| PPP | 63.41 | 72.06 | 67.74 | 0.36 | 0.735 |
| PPP+SS | 66.22 | 70.67 | 68.44 | 0.37 | 0.742 |
| PPP+ASA | 66.67 | 68.89 | 67.78 | 0.36 | 0.735 |
| PPP+SS+ASA | 67.11 | 69.78 | 68.44 | 0.37 | 0.746 |
