# Supplementary material for: In silico Platform for Prediction of N-, O- and C-Glycosites in Eukaryotic Protein Sequences
Source: PLoS One. 2013 Jun 28;8(6):e67008. doi: 10.1371/journal.pone.0067008 (PMC3695939; doi:10.1371/journal.pone.0067008)
Supplement: Table S17 — Performance of SVM using conserved sequon information along with BPP, CPP or PPP as input features for prediction of N-linked glycosylation sites using Sequon datasets. (DOCX) [file pone.0067008.s021.docx]

**Table S17:** Performance of SVM using conserved sequon information along with BPP, CPP or PPP as input features for prediction of N-linked glycosylation sites using Sequon datasets.

| Prediction Feature | Sensitivity | Specificity | Accuracy | MCC |
| --- | --- | --- | --- | --- |
| BPP | 65.11 | 69.68 | 67.37 | 0.35 |
| CPP | 60.26 | 57.52 | 58.91 | 0.18 |
| PPP | 60.58 | 57.48 | 59.05 | 0.18 |
